# New Ionic Carbosilane Dendrons Possessing Fluorinated Tails at Different Locations on the Skeleton

**DOI:** 10.3390/molecules25040807

**Published:** 2020-02-13

**Authors:** Gabriel Mencia, Tania Lozano-Cruz, Mercedes Valiente, Javier de la Mata, Jesús Cano, Rafael Gómez

**Affiliations:** 1 Departamento de Química Orgánica y Química Inorgánica, IQAR, Universidad de Alcalá, Campus Universitario, Alcalá de Henares, 28805 Madrid, Spain; gabi.men.ber@gmail.com (G.M.); tania.lozano@uah.es (T.L.-C.); javier.delamata@uah.es (J.d.l.M.); jesus.cano@uah.es (J.C.); 2 Networking Research Center on Bioengineering, Biomaterials and Nanomedicine (CIBER-BBN), 28029 Madrid, Spain; 3 Ramón y Cajal Health Research Institute (IRYCIS), IRYCIS, 28034 Madrid, Spain; 4 Departamento de Química Analítica, Química Física e Ingeniería Química, IQAR, Universidad de Alcalá Campus Universitario, Alcalá de Henares, 28805 Madrid, Spain; mercedes.valiente@uah.es

**Keywords:** carbosilane, dendrimer, fluorine, micelle

## Abstract

The fluorination of dendritic structures has attracted special attention in terms of self-assembly processes and biological applications. The presence of fluorine increases the hydrophobicity of the molecule, resulting in a better interaction with biological membranes and viability. In addition, the development of ^19^F magnetic resonance imaging (^19^F-MRI) has greatly increased interest in the design of new fluorinated structures with specific properties. Here, we present the synthesis of new water-soluble fluorinated carbosilane dendrons containing fluorinated chains in different positions on the skeleton, focal point or surface, and their preliminary supramolecular aggregation studies. These new dendritic systems could be considered as potential systems to be employed in drug delivery or gene therapy and monitored by ^19^F-MRI.

## 1. Introduction

Since their discovery, the well-defined architecture of dendrimers has promoted their use thanks to their highly multifunctional skeleton, which not only allows for the concentration of a high number of units of interest in the same structure but also permits fragments of a different nature to be introduced, combining both properties. Therefore, the introduction of different types of fragment enables change in the physicochemical properties of the dendritic structure, yielding molecules with relevance in diverse fields such as catalysis [1], material science [2] or biomedicine [3]. 

Taking advantage of these structural features of dendrimers, fluorination has attracted the attention of researchers to explore catalytic [4] or electronic properties [5], although special interest has been shown in supramolecular chemistry [6] or biology [7] in recent years. Moreover, the presence of fluorine increases lipophilicity, changing the amphiphilic character. Fluorinated dendritic systems of different topologies have been shown to form micelles with encapsulation ability [8,9] or dendrimersomes with thicknesses similar to biological membranes [10,11]. In addition, the presence of fluorine stimulates the interaction between biomembranes, imparting a favorable impact on biocompatibility. In this sense, fluorinated naked dendrimers or supramolecular dendritic aggregates with improved drug or gene delivery activity and viability have been designed, producing better results than their perhydrogenated counterparts [12,13]. Extensive research on fluorine-containing PAMAM dendritic systems in biomedical applications has been described elsewhere. However, carbosilane dendrimers remain scarcely explored in this field, with few examples of high generation fluorine-doped carbosilane dendrimers [14,15,16] or dendrons [17] reported for other applications.

Fluorine is largely absent in biological systems (with just small traces found in bones or teeth); however, with its 100% natural abundance, high receptivity (83% with respect to ^1^H), equivalent magnetic resonance to ^1^H (94%) and a broad-spectrum range, these nuclei are promising for use in singular magnetic resonance imaging (MRI) techniques [18]. Fluorinated dendrimers could be monitored by ^19^F-MRI to create personalized therapy, although aqueous solubility with a high stability would be required for this purpose. Consequently, the design of dendritic systems for this application remains a difficult task and the synthesis of adequate fluorine-containing dendrimers with specific MRI properties remains a challenge. 

Herein, we present the synthesis of new water-soluble dendrons functionalized with fluorinated chains at the periphery or focal point, employing a simple synthetic route that makes use of perfluorocarbon compounds. Ammonium or sulfonate groups are used to provide aqueous solubility. In addition, differences in the presence of an ester or amide bond as a linker are discussed and self-assembly properties are evaluated.

## 2. Results and Discussion

**Figure 1:**
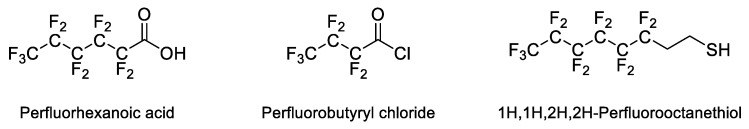
Fluorinated reagents employed in the fluorine-containing dendrons synthesis.

Dendron topology was selected to control the homogeneity of the structure and particularly taking into account the possible dendronization processes over other platforms, such as nanoparticles, polymers or drugs. In addition, perfluorinated derivatives were chosen based on their well-known chain length–bioaccumulation relationship—the limit was established in six perfluorinated carbons to be considered as non-bioaccumulative compounds [19,20,21]. All employed fluorinated precursors for the dendron functionalization used in this work and are shown in Figure 1.

### 2.1. Dendrons with Perfluorinated Fatty Acids at the Focal Point

The first synthetic strategy was directed toward the functionalization of the dendron focal point. Considering the fluorinated precursors, the ester and the amide functional groups were selected to act as linkers between the dendritic system and the perfluorinated chain. Furthermore, ammonium groups were used in the periphery based on their previously reported capacity to bind nucleic material of analogous perhydrogenated dendrons [22].

**Scheme 1:**
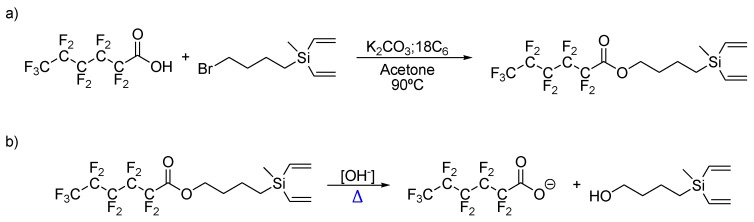
(**a**) Perfluorinated chain introduction at the focal point of the dendron; (**b**) basic hydrolysis reaction of the ester bond (see Table 1).

The initial approach to synthetize dendrons containing an ester bond as the anchor point involved an esterification reaction between perfluorhexanoic acid and dendrons with a bromine atom at the focal point and vinyl peripheral groups (BrG_1_V_2_) [23] in general basic pH conditions (K_2_CO_3_) (Scheme 1a). Monitoring by ^1^H-NMR confirmed the formation of the expected product, although some hydrolysis side products (ester bond disruption) were always observed; increasing the reaction time gave the perfluorinated carboxylate compound and a dendron containing a hydroxylic group at the focal point (Scheme 1b). The signals in ^1^H-NMR of different methylene groups identified in the spectrum are detected at 3.44 ppm for the initial BrCH_2_– unit, 4.51 ppm for the ester group R^F^COOCH_2_– and 3.52 ppm for the alcohol derivative HOCH_2_– (Figure S1). ^19^F-NMR experiments also confirmed the presence of three fluorinated species showing resonances for the –CF_2_CO_2_– fragment at −119.9 ppm of the starting precursor perfluorohexanoic acid, −118.2 ppm for carboxylate derivative, and −116.3 ppm for the compound containing the ester bond (Figure S2). The quick saponification process is attributed to the presence of fluorine atoms in the substituents of the carbonyl group, which exert a strong electron withdrawing effect. Consequently, the carboxylate derivative formed is quite stable and stimulates the complete hydrolysis of the ester bond.

Aiming to reduce the rate of the basic hydrolysis reaction or to fully prevent it, several modifications of the synthetic protocol were studied, including the nature of basic agent, the number of equivalents of the base employed and the temperature. The conditions tested are shown in Table 1. Unfortunately, it was not possible to obtain the pure esterification product, but some conclusions can be drawn: (i) high temperature and basic conditions are necessary for both esterification and hydrolysis reactions and (ii) the nature of the base and stoichiometry affect the process.

**Scheme 2:**
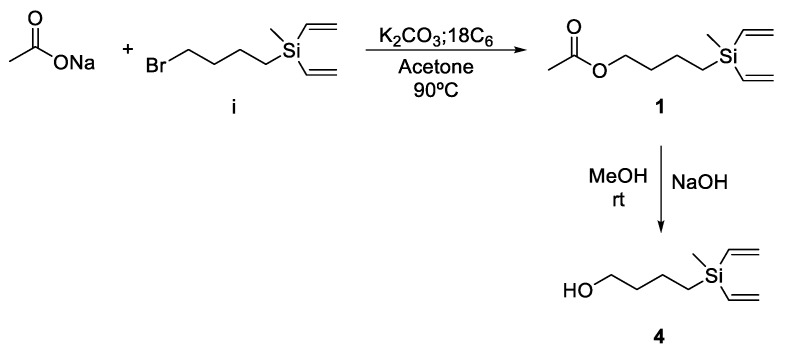
Synthetic route employed to prepare dendrons containing a hydroxyl group at the focal point illustrated for the first generation.

Keeping in mind that the basic conditions are responsible for the disruption of the ester bond, a new strategy was designed to obtain the desired dendrons with an ester group as the anchor point. Here, new dendrons with a hydroxyl unit at the focal point, HOGnVm, which can react with acyl halides, were necessary for the synthesis. These dendritic wedges were synthetized in two steps from BrGnVm (n = 1, m = 2 (**i**); n = 2, m = 4 (**ii**); n = 3, m = 8 (**iii**)) dendrons, as illustrated in Scheme 2. 

The first step entailed coupling the sodium acetate to the dendrons **i**–**iii** to give the dendritic systems CH_3_CO_2_GnVm (n = 1, m = 2 (**1**); n = 2, m = 4 (**2**); n = 3, m = 8 (**3**)). The reaction was monitored by ^1^H-NMR experiments until complete, when the resonance at 4.00 ppm was attributed to the methylene unit attached to oxygen atom –CO_2_CH_2_–. In the ^13^C-NMR spectrum, the signal assigned to the same fragment –CO_2_CH_2_– was detected at 64.1 ppm while the carbonyl group resonance was distinguished at 171.0 ppm. The next step was the saponification reaction of the ester bond in basic media (NaOH) to obtain the hydroxyl dendritic derivatives HOGnVm (n = 1, m = 2 (**4**); n = 2, m = 4 (**5**); n = 3, m = 8 (**6**)). Again, the reaction was monitored by NMR experiments, with resonances for the HOCH_2_- fragment at 3.59 ppm in ^1^H-NMR and 62.5 ppm in ^13^C-NMR. 

**Scheme 3:**
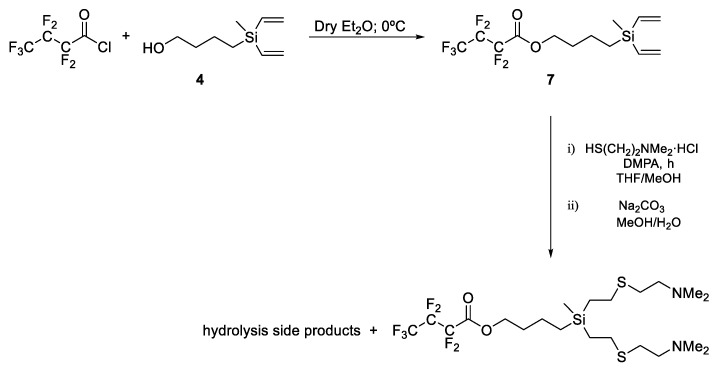
Alternative route to the preparation of fluorinated dendrons with an ester bond.

The preparation of cationic fluorinated dendrons with an ester bond through this synthetic route was initially studied for the first generation dendron **4** and perfluorobutyryl chloride (Scheme 3). After 2 hours, the reaction was monitored by ^1^H-NMR, confirming the formation of the desired product C_3_F_7_CO_2_G_1_V_2_ (**7**). Characteristic signals of this compound were observed at 4.37 ppm for –CO_2_CH_2_– in the ^1^H-NMR spectrum (Figure S3), 68.2 ppm and 158.2 ppm for the methylene unit and the carbonyl group in the same fragment in the ^13^C{^1^H}-NMR spectrum (Figure S4), and −127.2 ppm (CF_3_CF_2_CF_2_CO_2_–), −119.5 ppm (CF_3_CF_2_CF_2_CO_2_–) and −81.0 ppm (CF_3_CF_2_CF_2_CO_2_–) in ^19^F-NMR experiments (Figure S5). Chemical shift assignments for the carbon atoms in the fluorinated chain were determined by ^13^C{^19^F}-NMR (Figure S6), with the coupling constants; ^1^J(^13^C-^19^F) around 280 Hz and ^3^J(^13^C-^19^F) around 30 Hz were also obtained. In this case, no side products from hydrolysis were observed and ^1^H-NMR stability experiments revealed that dendritic wedge **7** was stable for months under an inert atmosphere. A thiol-ene reaction was then employed to introduce cationic groups at the periphery using 2-(dimethyl)ethanethiol hydrochloride. Unfortunately, side products from the hydrolysis reaction were observed again, probably due to a combination of several factors such as an amino group-containing reagent acting as base and ultraviolet radiation, which results in the overheating of the solution.

**Scheme 4:**
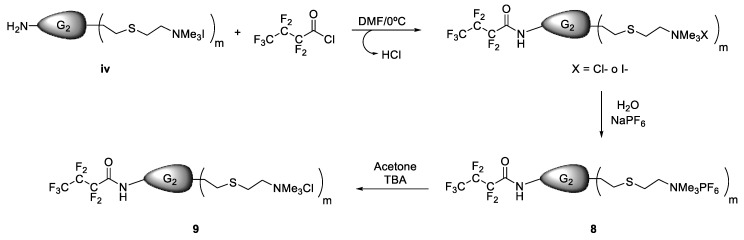
Synthetic route for cationic amide bond-containing dendrons with a perfluorinated chain at the focal point.

**Figure 2:**
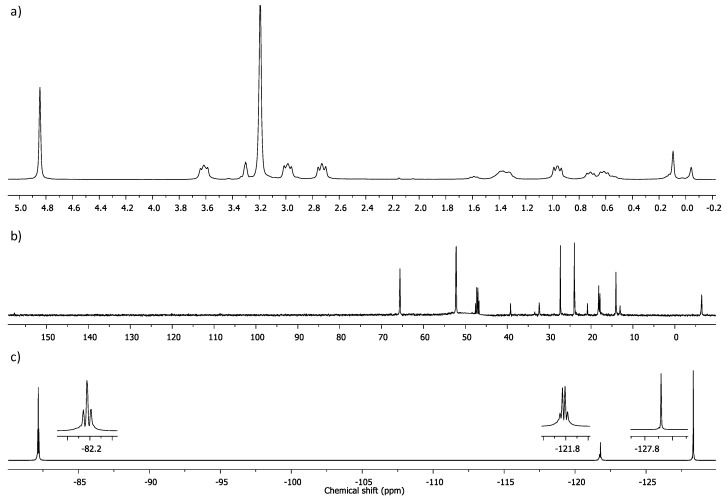
(**a**) ^1^H-NMR; (**b**) ^13^C{^1^H}-NMR and (**c**) ^19^F-NMR spectra of compound **9** in CD_3_OD.

Considering the low stability of the ester bond in the presence of fluorine atoms with respect to the analogous hydrocarbon compounds, efforts to obtain fluorinated amphiphilic dendritic wedges were centred on the amide linker strategy. In this case, the selected precursors were ammonium-functionalized dendrons with an amine group at the focal point NH_2_G_2_(SNMe_3_I)_4_ (**iv**) [24] because it was not possible to prepare the amine derivative analogous to compound **4** in good yields through the Gabriel synthesis for primary amines. The formation of the amide bond was carried out by employing perfluorobutyryl chloride and dry DMF as the solvent at 0 °C (Scheme 4). The colorimetric Kaiser test confirmed the absence of free amine groups in the reaction medium. Allylamine was then added to the solution to consume all of the perfluorinated compound. The presence of a new amide derivative was also confirmed in situ by ^1^H-NMR experiments showing a resonance at 3.30 ppm for –CONHCH_2_– (Figure 2). 

A mixture of counter anions (Cl^−^ and I^−^) was obtained during the amide bond formation due to HCl release in this process. In order to obtain the chloride derivative as the only product, a counter-ion exchange was carried out. Firstly, iodide (I^−^) and chloride (Cl^−^) ions were replaced by a hexafluorophosphate (PF_6_^−^) anion by adding an aqueous solution of sodium hexafluorophosphate (NaPF_6_) to produce C_3_F_7_CONHG_2_(SNMe_3_PF_6_)_4_ (**8**). This process modified the dendron solubility in water, a necessity to accomplish a second derivatization. Hence, tetrabutylammonium chloride was added to a solution of compound **8** in acetone to give the amphiphilic dendron C_3_F_7_CONHG_2_(SNMe_3_Cl)_4_ (**9**). NMR experiments (^1^H, ^13^C, ^19^F) show similar patterns for compounds **8** and **9** (Figure 2). The only difference was observed in the ^19^F-NMR spectrum where the PF_6_^−^ counter-anion affords a resonance at −73.5 ppm along with the resonances for the perfluorinated unit, a singlet at −128.3 ppm (CF_3_CF*_2_*CF_2_CO_2_–), a quadruplet at −121.8 ppm (CF_3_CF_2_CF*_2_*CO_2_–) and a triplet at −82.2 ppm (CF_3_CF_2_CF_2_CO_2_–). In our hands, and due to the low solubility of **9** and the low relaxing times of carbon atoms, neither the carbons in the fluorinated chain nor in the carbonyl group were observed in the proton or fluorine decoupling ^13^C-NMR experiments. For a representative structure of compound **9** see below (vide infra).

#### Self-Assembly Assay of Dendron C_3_F_7_CONHG_2_(SNMe_3_Cl)_4_ (**9**)

**Figure 3:**
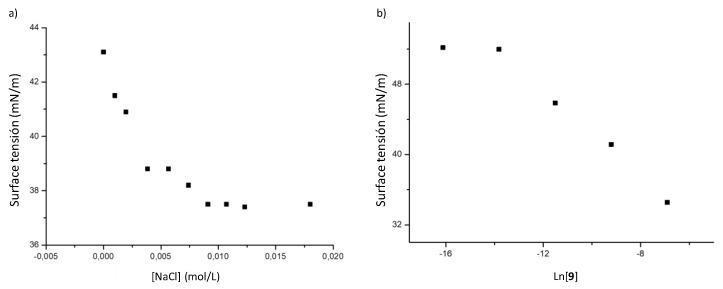
(**a**) Surface tension measurement of a 1 mM solution of dendron C_3_F_7_CONHG_2_(SNMe_3_Cl)_4_ (**9**) at increasing concentration of NaCl; (**b**) Surface tension measurements of dendritic wedge C_3_F_7_CONHG_2_(SNMe_3_Cl)_4_ (**9**) at the ionic strength of 12 mM of NaCl.

Looking for micelle formation, the amphiphilic behaviour of fluorinated compound **9** was studied by surface tension measurements using the Du Noüy ring method. The experiment was carried out in an aqueous solution with an ionic strength because the presence of salts in the medium has been shown to reduce the critical micellar concentration (CMC) of compounds [25,26]. To determine the optimum salt concentration, a solution of dendron **9** (1 mM) in deionized water was incubated with increasing concentrations of salts. The measurement at 30 °C showed stable surface tension values starting from 10 mM of NaCl (Figure 3a). For that reason, the selected salt concentration to perform the measurements with dendrons was 12 mM.

A CMC determination from an aqueous solution of dendritic wedge **9** was studied by increasing the concentration of dendrons from 10^−7^ to 10^−3^ M (Figure 3b). Regrettably, it was not possible to reach stable values of surface tension in the range studied, which indicated that the CMC value was above 1 mM. Considering the equivalency rule 1 CF_2_ ≈ 1.5 CH_2_ [27], the lipophilicity of the perfluorinated chain in compound **9** (4–5 carbon atoms) should be near to a hydrocarbon fragment with six carbon atoms. In that sense, the result found is in agreement with that described in the literature for analogous dendrons with a hexanoate unit at the focal point—this does not generate micelles at concentrations lower than 1 mM [22]. However, it is possible to conclude that the surface activity of **9** is higher than that of the analogous hydrocarbon fatty acid-containing dendrons, as evidenced by the greater reduction in the surface tension of water at 1 mM of compound **9** to 34.6 mN/m with respect to the analogous hydrocarbon dendron with a sixteen carbon atom chain (PalG_2_(SNMe_3_I)_4_) of 45.0 mN/m [22].

### 2.2. Dendrons with Perfluorinated Chains at the Periphery

The second synthetic strategy was the functionalization of the dendron periphery. The introduction of fluorine-containing branches on the dendritic surface was performed through thiol-ene click reactions for second- and third-generation carbosilane dendrons. Each generation contains different proportions of the perfluorocarbon chain because water solubility could be affected by an incorrect hydrophilic–lipophilic balance in the structure. For that reason, second-generation dendrons were designed to contain just one perfluorinated chain while third-generation compounds include two. In these cases, fluorinated fragments were introduced at the periphery to keep the orthogonal and reactive group at the focal point available for dendronization processes. Sulfonate groups were selected as ionic groups for these systems rather than ammonium groups, given that anionic units contribute to higher water solubility and lower toxicity values. 

**Scheme 5:**
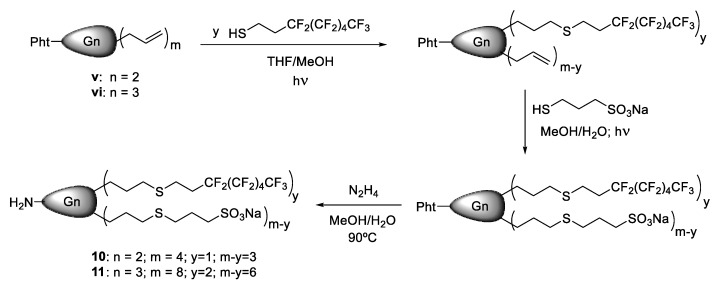
Synthetic route for the preparation of anionic dendrons with a perfluorinated chain on the surface and an amino group at the focal point.

As shown in Scheme 5, this synthetic route involves the initial incorporation of 1H, 1H, 2H,2H-perfluorooctanethiol over superficial allyl groups of the dendritic precursor with the phtalimide unit at the focal point PhGnAm (n = 2, m = 4 (**v**); n = 3, m = 8 (**vi**)) [28] in a ratio 1:y, yielding statistical decoration in the dendrimers due to the equal reactivity of the allyl groups [21], and the subsequent functionalization of the rest of allyl groups with sodium 3-mercapto-1-propanesulfonate. The thiol-ene functionalization of the surface was carried out in 4 h by a photocatalytic process using 2,2-dimethoxy-2-phenylacetophenone (DMPA) as photoinitiator. Final deprotection of the focal point with hydrazine hydrochloride at 90 °C affords the desired derivatives **10** and **11**. 

**Figure 4:**
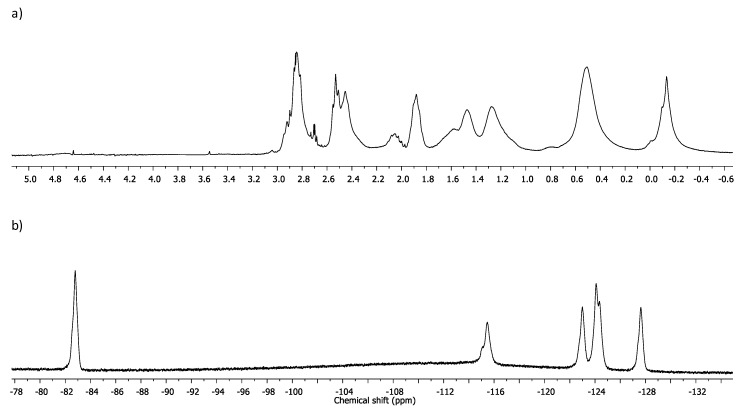
(**a**) ^1^H-NMR and (**b**) ^19^F-NMR spectra of compound **10** in D_2_O.

**Figure 5:**
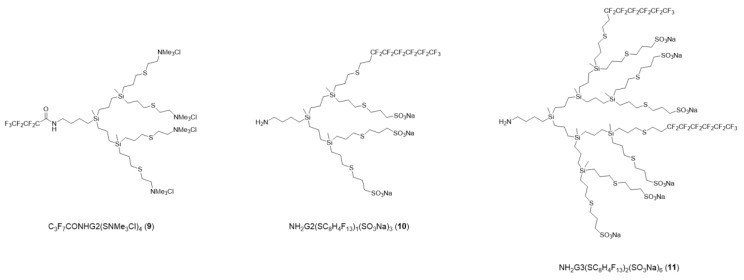
Representative structures of fluorinated carbosilane dendrons.

The complete derivatization of the allyl unit was confirmed by the disappearance of resonances in the range 4.50–6.00 ppm in the ^1^H-NMR spectrum. New characteristic signals are located at 2.04 and 2.72 ppm, corresponding to SCH_2_CH_2_(C_6_F_13_) and SCH_2_CH_2_(C_6_F_13_), respectively (Figure 4). Resonances of –SCH_2_CH_2_CH_2_SO_3_Na appear at 1.89 ppm for the internal methylene group, 2.52 ppm for –CH_2_– unit attached to the thioeter group and 2.85 ppm for the –CH_2_SO_3_Na methylene fragment, as described elsewhere [29]. In addition, the ^19^F-NMR spectrum shows resonances at −85.8 ppm for the –CF_3_ unit and −115.5, −123.0, −124.2 and −127.7 ppm for –CF_2_− fragments. Representative structures of compounds 10 and 11 aredrawn in Figure 5.

## 3. Conclusions

A simple and rapid procedure has been designed to prepare ionic carbosilane dendrons containing fluorinated units at two different positions in the skeleton. The first strategy incorporates fluorine-containing chains at the focal point of cationic dendrons producing amphiphilic dendrons. Although the amphiphilic compound **9** does not show supramolecular assembly formation in the range 10^−3^–10^−7^ M, the synthetic route opens the door to extrapolate this procedure to dendritic systems with appropriate hydrophilic–lipophilic balances that give rise to micelles. This property can be obtained by increasing both the length of the perfluorinated unit at the focal point and the generation of the dendron. The second approach affords anionic dendritic wedges with fluorinated fragments at the periphery through thiol-ene click reactions. This family of compounds contains a reactive amine group at the focal point available to be conjugated to nanostructured materials such as nanoparticles or biomolecules through so-called dendronization processes. Both strategies produce systems with fluorine units in the dendritic structure which may create delivery systems with the potential to provide imaging agents for innovative ^19^F-MRI.

## 4. Materials and Methods 

### 4.1. Materials

Solvents and reagents were obtained from commercial sources. Et_2_O and DMF were appropriately dried before use, while other reagents were employed without further purification. Dendritic precursors BrGnVm (**i**–**iii**) [23], NH_2_G_2_(SNMe_3_I)_4_ (**iv**) [24], PhtG_n_A_m_ (**v**–**vi**) were prepared as described elsewhere [28]. Click reactions (thiol-ene addition) were carried out employing a HPK 125 W mercury lamp (Heraeus Nobleligth; λ_max_ = 365 nm). NMR spectra were recorded on a Varian Unity VXR-300, Varian Mercury 300 or Varian 500 Plus instruments (Agilent Technologies, Palo Alto, CA, USA). Mass spectra were recorded on an Agilent 6210 TOF LC/MS instrument (LECO Instrumentos S.L, Madrid, Spain) for ESI–TOF. Surface tension was measured on a Lauda tensiometer TE 2/3 (LAUDA Measuring Instruments, Königshofen, Germany) with Pt/Ir ring.

### 4.2. Surface Tension Measurements

The samples were prepared using Milli-Q water as the solvent to give solutions in a concentration range of 0.1–1000 μM. In the case of salt solutions, NaCl was dissolved in Milli-Q water until reaching a final concentration which was previously determined for each compound (12 or 20 mM). This solution was used in the preparation of samples following the same procedure as that described above. The surface tension of dendron solutions was determined at 30.0 ± 0.1 °C as a function of the concentration using the ring method with a standard deviation lower than 0.1 mN/m. Using the least-squares method, straight lines were fitted in the graphic surface tension versus the logarithm of concentration curves, where the CMC values correspond to the sharp break point from both lines.

### 4.3. Experimental Data 

CH_3_CO_2_G_1_V_2_ (**1**)

A solution of dendron BrG_1_V_2_ (**i**) [23] (1.000 g; 4.29 mmol) and sodium acetate (0.530 g; 6.46 mmol) in acetone (50 mL) was heated at 90 °C for 24 h in an ampoule with a high vacuum valve in the presence of K_2_CO_3_ (1.200 g; 8.68 mmol) and 18-crown-6 (0.088 g; 0.33 mmol). Then, volatiles were removed under vacuum and the crude product was extracted into Et_2_O (3 × 20 mL) and dried over MgSO_4_. During the drying period, traces of silica gel were added to eliminate 18-crown-6 and the desired dendron was obtained as an orange oil in excellent yield (92%). 

^1^H-NMR (CDCl_3_): δ (ppm) 0.08 (s, 3H, –Si(CH*_3_*)), 0.61 (m, 2H, –OCH_2_CH_2_CH_2_CH*_2_*Si–), 1.34 (m, 2H, –OCH_2_CH_2_CH*_2_*CH_2_Si–), 1.60 (m, 2H, –OCH_2_CH_2_CH_2_CH*_2_*Si–), 1.98 (s, CH*_3_*COOR), 4.00 (t, 2H, –OCH*_2_*CH_2_CH_2_CH_2_Si–), 5.66 (m, 2H, –SiCH=CH_2_), 6.02 (m, 4H, –SiCH=CH*_2_*).

^13^C-NMR: (CDCl_3_): δ (ppm) −5.49 (–Si(CH_3_)), 13.5 (–OCH_2_CH_2_CH_2_CH_2_Si–), 20.0 (–OCH_2_CH_2_CH_2_CH_2_Si–), 20.9 (CH_3_COOR), 32.1 (–OCH_2_CH_2_CH_2_CH_2_Si–), 64.1 (–OCH_2_CH_2_CH_2_CH_2_Si–), 132.9 (–SiCH=CH_2_), 136.6 (–SiCH=CH_2_), 171.0 (CH_3_COOR).

MS: [M + H]^+^ = 213.1341 Da (calcd. = 213.1305 Da).

Elemental analysis: C_11_H_20_O_2_Si (212.36 g/mol): calcd. = C, 62.21; H, 9.49; O, 15.07; Si, 13.22. Found. = C, 62.46; H, 9.33.

CH_3_CO_2_G_2_V_4_ (**2**)

Compound **2** was prepared using the protocol described for **1**, starting from BrG_2_V_4_ (**ii**) [23] (1.0 g; 2.18 mmol), sodium acetate (0.259 g; 3.16 mmol), K_2_CO_3_ (0.614 g; 4.44 mmol) and 18-crown-6 (0.063 g; 0.24 mmol). Yield: 98%.

^1^H-NMR: (CDCl_3_): δ (ppm) −0.07 (s, 3H, –Si(CH_3_)), 0.09 (s, 6H, –Si(CH_3_)), 0.56 (m, 6H, –OCH_2_CH_2_CH_2_CH_2_Si–, –SiCH_2_CH_2_CH_2_SiVinyl), 0.67 (m, 4H, –SiCH_2_CH_2_CH_2_SiVinyl), 1.35 (m, 6H, –OCH_2_CH_2_CH_2_CH_2_Si–, –SiCH_2_CH_2_CH_2_Si–), 1.63 (m, 2H, –OCH_2_CH_2_CH_2_CH_2_Si–), 2.00 (s, 3H, CH_3_COOR), 3.99 (t, 2H, –OCH_2_CH_2_CH_2_CH_2_Si–), 5.66 (m, 4H, –SiCH=CH_2_), 6.05 (m, 8H, –SiCH=CH_2_).

^13^C-NMR (CDCl_3_): δ (ppm) −5.53 (–Si(CH_3_)), −5.41 (–Si(CH_3_), 13.6 (–OCH_2_CH_2_CH_2_CH_2_Si–), 18.2-18.5 (–SiCH_2_CH_2_CH_2_Si–), 20.2 (–OCH_2_CH_2_CH_2_CH_2_Si–), 21.0 (CH_3_COOR), 32.1 (–OCH_2_CH_2_CH_2_CH_2_Si–), 64.2 (–OCH_2_CH_2_CH_2_CH_2_Si–), 132.7 (–SiCH=CH_2_), 136.8 (–SiCH=CH_2_), 170.9 (CH_3_COOR).

MS: [M + H]^+^ = 437.2718 Da (calcd. = 437.2722 Da).

Elemental analysis: C_23_H_44_O_2_Si_3_ (436.86 g/mol): calcd. = C, 63.24; H, 10.15; O, 7.32; Si, 19.29. Found. = C, 63.11; H, 9.97.

CH_3_CO_2_G_3_V_8_ (**3**)

Compound **3** was prepared using the protocol described for **1**, starting from BrG_3_V_8_ (**iii**) [23] (1.0 g; 1.10 mmol), sodium acetate (0.141 g; 1.72 mmol), K_2_CO_3_ (0.310 g; 2.24 mmol) and 18-crown-6 (0.027 g; 0.10 mmol). Yield: 98%.

^1^H-NMR: (CDCl_3_): δ (ppm) −0.09 (s, 9H, –Si(CH_3_)), 0.13 (s, 12H, –Si(CH_3_)), 0.55 (m, 18H, –OCH_2_CH_2_CH_2_CH_2_Si–, –SiCH_2_CH_2_CH_2_Si–, –SiCH_2_CH_2_CH_2_SiVinyl), 0.71 (m, 8H, –SiCH_2_CH_2_CH_2_SiVinyl), 1.33 (m, 14H, –OCH_2_CH_2_CH_2_CH_2_Si–, –SiCH_2_CH_2_CH_2_Si–), 1.65 (m, 2H, –OCH_2_CH_2_CH_2_CH_2_Si–), 2.04 (s, 3H, CH_3_COOR), 4.05 (t, 2H, –OCH_2_CH_2_CH_2_CH_2_Si–), 5.68 (m, 8H, –SiCH=CH_2_), 6,07 (m, 16H, –SiCH=CH_2_).

^13^C-NMR: (CDCl_3_): δ (ppm) -5.53 (–Si(CH_3_)), −5.41 (–Si(CH_3_)), 13.5 (–OCH_2_CH_2_CH_2_CH_2_Si–), 18.3-18.7 (–SiCH_2_CH_2_CH_2_Si–), 20.2 (–OCH_2_CH_2_CH_2_CH_2_Si–), 20.9 (CH_3_COOR), 32.3 (–OCH_2_CH_2_CH_2_CH_2_Si–), 64.5 (–OCH_2_CH_2_CH_2_CH_2_Si–), 132.4 (–SiCH=CH_2_), 136.7 (–SiCH=CH_2_), 171.0 (CH_3_COOR).

MS: [M + H]^+^ = 885.5593 Da (calcd. = 885.5555 Da).

Elemental analysis: C_47_H_92_O_2_Si_7_ (885.85 g/mol): calcd. = C, 63.73; H, 10.47; O, 3.61; Si, 22.19. Found. = C, 63.49; H, 10.14.

HOG_1_V_2_ (**4**)

A methanolic solution of compound **1** (0.80 g; 3.77 mmol) and NaOH (0.30 g; 7.50 mmol) was stirred at room temperature for 30 min. Afterwards, the solvent was removed under reduced pressure and the crude product was extracted into Et_2_O and dried over MgSO_4_, and the desired product was obtained as an orange oil in excellent yield (86%).

^1^H-NMR (CDCl_3_): δ (ppm) 0.13 (s, 3H, –Si(CH_3_)), 0.65 (m, 2H, –OCH_2_CH_2_CH_2_CH_2_Si–), 1.38 (m, 2H, –OCH_2_CH_2_CH_2_CH_2_Si–), 1.57 (m, 2H, –OCH_2_CH_2_CH_2_CH_2_Si–), 1,99 (s, 1H, –OH), 3.59 (t, 2H, –OCH_2_CH_2_CH_2_CH_2_Si–), 5.68 (m, 2H, –SiCH=CH_2_), 6.05 (m, 4H, –SiCH = CH_2_). 

^13^C-NMR (CDCl_3_): δ (ppm) −5.29 (–Si(CH_3_)), 13.9 (–OCH_2_CH_2_CH_2_CH_2_Si–), 20.0 (–OCH_2_CH_2_CH_2_CH_2_Si–), 36.5 (–OCH_2_CH_2_CH_2_CH_2_Si–), 62.5 (–OCH_2_CH_2_CH_2_CH_2_Si–), 132.7 (–SiCH=CH_2_), 136.7 (–SiCH=CH_2_).

MS: [M + H]^+^ = 171.1214 Da (calcd. = 171.1200 Da).

Elemental analysis: C_9_H_18_OSi (170.33 g/mol): calcd. = C, 63.47; H, 10.65; O, 9.39; Si, 16.49. Exp. = C, 63.34; H, 10.55.

HOG_2_V_4_ (**5**)

Compound **5** was prepared using the protocol described for **4**, starting from CH_3_CO_2_G_2_(V)_4_ (**2**) (0.800 g; 1.83 mmol) and NaOH (0.153 mg; 3.83 mmol). Yield: 89%.

^1^H-NMR (CDCl_3_): δ (ppm) −0.08 (s, 3H, –Si(CH_3_)), 0.13 (s, 6H, –Si(CH_3_)), 0.49 (m, 2H, –OCH_2_CH_2_CH_2_CH_2_Si–), 0.56 (m, 4H, –SiCH_2_CH_2_CH_2_SiVinyl), 0.70 (m, 4H, –SiCH_2_CH_2_CH_2_SiVinyl), 1.35 (m, 6H, –OCH_2_CH_2_CH_2_CH_2_Si–, –SiCH_2_CH_2_CH_2_Si–), 1.58 (m, 2H, –OCH_2_CH_2_CH_2_CH_2_Si–), 1,99 (s, 1H, –OH), 3.62 (t, 2H, –OCH_2_CH_2_CH_2_CH_2_Si–), 5.68 (m, 4H, –SiCH=CH_2_), 6.07 (m, 8H, –SiCH=CH_2_). 

^13^C-NMR (CDCl_3_): δ (ppm) −5.42 (–Si(CH_3_)), −5.28 (–Si(CH_3_)), 13.9 (–OCH_2_CH_2_CH_2_CH_2_Si–), 18.2–18.4 (–SiCH_2_CH_2_CH_2_Si–), 20.2 (–OCH_2_CH_2_CH_2_CH_2_Si–), 36.2 (–OCH_2_CH_2_CH_2_CH_2_Si–), 62.6 (–OCH_2_CH_2_CH_2_CH_2_Si–), 132.8 (–SiCH=CH_2_), 136.7 (–SiCH=CH_2_).

MS: [M + H] ^+^ = 395.2586 uma (calcd. = 395.2616 uma).

Elemental analysis: C_21_H_42_OSi_3_ (394.82 g/mol): calcd. = C, 63.88; H, 10.72; O, 4.05; Si, 21.34. Exp. = C, 63.67; H, 10.58.

HOG_3_V_8_ (**6**)

Compound **6** was prepared using the protocol described for **4**, starting from CH_3_CO_2_G_3_(V)_8_ (**3**) (0.800 mg; 0.90 mmol), NaOH (0.790 mg; 1.98 mmol). Yield: 88%.

^1^H-NMR: (CDCl_3_): δ (ppm) −0.07 (s, 6H, –Si(CH_3_)), −0.05 (s, 3H, –Si(CH_3_)), 0.15 (s, 12H, –Si(CH_3_)–), 0.57 (m, 18H, –OCH_2_CH_2_CH_2_CH_2_Si–, –SiCH_2_CH_2_CH_2_Si–, –SiCH_2_CH_2_CH_2_SiVinyl), 0.72 (m, 8H, –SiCH_2_CH_2_CH_2_SiVinyl), 1.37 (m, 14H, –OCH_2_CH_2_CH_2_CH_2_Si–, –SiCH_2_CH_2_CH_2_Si–), 1.60 (m, 2H, - OCH_2_CH_2_CH_2_CH_2_Si–), 1,99 (s, 1H, -OH), 3,65 (t, 2H, –OCH_2_CH_2_CH_2_CH_2_Si–), 5.70 (m, 8H, –SiCH=CH_2_), 6.08 (m, 16H, –SiCH=CH_2_). 

^13^C-NMR: (CDCl_3_): δ (ppm) −5.46 (–Si(CH_3_)), −5.29 (–Si(CH_3_)), 13.6 (–OCH_2_CH_2_CH_2_CH_2_Si–), 18.1–18.5 (–SiCH_2_CH_2_CH_2_Si–), 20.5 (–OCH_2_CH_2_CH_2_CH_2_Si–), 36.3 (–OCH_2_CH_2_CH_2_CH_2_Si–), 62.5 (–OCH_2_CH_2_CH_2_CH_2_Si–), 132.5 (–SiCH=CH_2_), 136.6 (–SiCH=CH_2_).

MS: [M + H]^+^ = 843.5416 Da (calcd. = 843.5449 Da).

Elemental analysis: C_45_H_90_OSi_7_ (843.81 g/mol): calcd. = C, 64.05; H, 10.75; O, 1.90; Si, 23.30. Exp. = C, 63.81; H, 10.93.

C_3_F_7_CO_2_G_1_V_2_ (**7**)

Under an inert atmosphere, perfluorobutyryl chloride (0.088 mL; 0.59 mmol) was slowly added to a solution of HOG_1_V_2_ (**4**) (0.100 mg; 0.59 mmol) in dry Et_2_O (50 mL) at 0 °C and was subjected to maintained stirring for 2 h. During this period, the HCl generated in situ as a side product was eliminated from the reaction medium by vacuum cycles every 30 min. After the completion time, the volatiles were removed, obtaining the fluorinated dendron as a colourless oil with an excellent yield (87%).

^1^H-NMR: (CDCl_3_): δ (ppm) 0.15 (s, 3H, –Si(CH_3_)), 0.67 (m, 2H, –OCH_2_CH_2_CH_2_CH_2_Si–), 1.43 (m, 2H, –OCH_2_CH_2_CH_2_CH_2_Si–), 1.77 (m, 2H, –OCH_2_CH_2_CH_2_CH_2_Si–), 4.37 (t, 2H, –OCH_2_CH_2_CH_2_CH_2_Si–), 5.70 (m, 2H, –SiCH=CH_2_), 6.06 (m, 4H, –SiCH=CH_2_).

^19^F-NMR (CDCl_3_): δ (ppm) −127.2 (s, 2F, CF_3_CF_2_CF_2_CO_2_R), −119.5 (c, 2F, CF_3_CF_2_CF_2_CO_2_R), −81.0 (t, 3F, CF_3_CF_2_CF_2_CO_2_R).

^13^C{^1^H}-NMR: (CDCl_3_): δ (ppm) −5.39 (–Si(CH_3_)), 13.6 (–OCH_2_CH_2_CH_2_CH_2_Si–), 19.9 (–OCH_2_CH_2_CH_2_CH_2_Si–), 31.6 (–OCH_2_CH_2_CH_2_CH_2_Si–), 68.2 (–OCH_2_CH_2_CH_2_CH_2_Si–), 107,6 (tt, CF_3_CF_2_CF_2_CO_2_R), 108.0 (tm, CF_3_CF_2_CF_2_CO_2_R), 117.4 (ct, CF_3_CF_2_CF_2_CO_2_R), 133.0 (–SiCH=CH_2_), 136.3 (–SiCH=CH_2_), 158.2 (t, –CF_2_CO_2_R).

^13^C{^19^F}-NMR: (CDCl_3_): δ (ppm) −5.57 (c, –Si(CH_3_)), 13.4 (t, –OCH_2_CH_2_CH_2_CH_2_Si–), 19.7 (t, –OCH_2_CH_2_CH_2_CH_2_Si–), 31.5 (t, –OCH_2_CH_2_CH_2_CH_2_Si–), 68.2 (t, –OCH_2_CH_2_CH_2_CH_2_Si–), 107.6 (CF_3_CF_2_CF_2_CO_2_R), 108.0 (CF_3_CF_2_CF_2_CO_2_R), 117.4 (CF_3_CF_2_CF_2_CO_2_R), 133.2 (t, –SiCH=CH_2_), 136.4 (d, –SiCH=CH_2_), 158.2 (–CF_2_CO_2_R).

MS: [Perfluorobutyric acid]^+^ = 213.9942 Da (calcd. = 213.9865 Da); [M + H-Perfluorobutanoyl]^+^ = 171.1187 Da (calcd. = 171.1200 Da).

Elemental analysis: C_13_H_17_F_7_O_2_Si (366.35 g/mol): calcd. = C, 42.62; H, 4.68; F, 36.30; O, 8.73; Si, 7.67. Exp. = C, 42.58; H, 4.69.

C_3_F_7_CONHG_2_(SNMe_3_PF_6_)_4_ (**8**)

Under an inert atmosphere, perfluorobutyryl chloride (0.022 mL; 0.15 mmol) was slowly added to a solution of NH_2_G_2_(SNMe_3_I)_4_ (**iv**) (0.200 g; 0.14 mmol) in dry DMF (20 mL) at 0 °C with stirring being maintained for 2 h. During this time, HCl generated in situ as a side product was eliminated from the medium by vacuum cycles every 30 min. The completion of the reaction was determined by a colorimetric Kaiser test. Allylamine (0.015 mL; 0.20 mmol) was then added at 0 °C and the reaction continued for 2 h. After this, the volatiles were removed, and the crude compound was redissolved in the minimum volume of hot acetonitrile and precipitated with Et_2_O and washed twice with the same solvent (2 × 10 mL). The product was then dissolved in water and an aqueous solution of NaPF_6_ was added (0.200 g; 1.19 mmol). The reaction was kept stirring for 2 h., filtered, washed twice with water and precipitated again with acetonitrile/Et_2_O. The amide-bond fluorinated dendron was obtained as a yellowish solid with a good yield (65%).

^1^H-NMR: (CD_3_OD): δ (ppm) 0.00 (s, 3H, –Si(CH_3_)), 0.12 (s, 6H, –Si(CH_3_)), 0.51–0.83 (m, 10H, –NCH_2_CH_2_CH_2_CH_2_Si–, –SiCH_2_CH_2_CH_2_Si–), 1.00 (m, 8H, –SiCH_2_CH_2_S-) 1.28-1.55 (m, 6H, –NCH_2_CH_2_CH_2_CH_2_Si–, –SiCH_2_CH_2_CH_2_Si–), 1.62 (m, 2H, –NCH_2_CH_2_CH_2_CH_2_Si–), 2.75 (m, 8H, –SiCH_2_CH_2_S–), 3.00 (m, 8H, –SCH_2_CH_2_N–), 3.20 (s, 36H, –N(CH_3_)_3_), 3.26 (t, 2H, –NCH_2_CH_2_CH_2_CH_2_Si–), 3.59 (m, 8H, –SCH_2_CH_2_N–).

^19^F-NMR: (CD_3_OD): δ (ppm) −128.4 (s, 2F, CF_3_CF_2_CF_2_CO_2_R), −121.8 (c, 2F, CF_3_CF_2_CF_2_CO_2_R), −82.5 (t, 3F, CF_3_CF_2_CF_2_CO_2_R), −73.5 (d, 24F, PF_6_^−^).

^13^C{^1^H}-NMR: (CD_3_OD): δ (ppm) -5.12 (–Si(CH_3_)), −4.91 (–Si(CH_3_)), 14.3 (–NCH_2_CH_2_CH_2_CH_2_Si–), 15.4 (–SiCH_2_CH_2_S–), 19.2-19.4 (–SiCH_2_CH_2_CH_2_Si–), 22.2 (–NCH_2_CH_2_CH_2_CH_2_Si–), 25.6 (–SiCH_2_CH_2_S–), 29.1 (–SCH_2_CH_2_N–), 33.7 (-NCH_2_CH_2_CH_2_CH_2_Si–), 40.6 (–NCH_2_CH_2_CH_2_CH_2_Si–), 53.4 (–N(CH_3_)_3_), 66.7 (–SCH_2_CH_2_N–).

MS: [M + H]^+^ = 1650.4057 Da (calcd. = 1650.4490 Da).

Elemental analysis: C_45_H_98_F_31_N_5_OP_4_S_4_Si_3_ (1650.65 g/mol): calcd. = C, 32.74; H, 5.98; F, 35.68; N, 4.24; O, 0.97; P, 7.51; S, 7.77; Si, 5.10. Exp. = C, 33.58; H, 6.03; N, 4.75; S, 7.38.

C_3_F_7_CONHG_2_(SNMe_3_Cl)_4_ (**9**)

A solution of tetrabutylammonium chloride (0.214 g; 0.15 mmol) in acetone was added to a solution of compound **8** (0.155 g; 93.9 µmol) in the same solvent (5 mL). The reaction was stirred for 2 h at room temperature. The solvent was then eliminated by filtration and the product washed twice with acetone to give compound **9** as a yellowish solid with an excellent yield (85%).

^1^H-NMR: (CD_3_OD): δ (ppm) 0.00 (s, 3H, –Si(CH_3_)), 0.13 (s, 6H, –Si(CH_3_)), 0.51–0.70 (m, 6H, –NCH_2_CH_2_CH_2_CH_2_Si–, –SiCH_2_CH_2_CH_2_Si–), 0.75 (m, 4H, –CH_2_SiCH_2_CH_2_S–), 1.00 (m, 8H, –SiCH_2_CH_2_S-) 1.27–1.53 (m, 6H, –NCH_2_CH_2_CH_2_CH_2_Si–, –SiCH_2_CH_2_CH_2_Si–), 1.62 (m, 2H, –NCH_2_CH_2_CH_2_CH_2_Si–), 2.77 (m, 8H, –SiCH_2_CH_2_S–), 3.02 (m, 8H, –SCH_2_CH_2_N–), 3.23 (s, 36H, –N(CH_3_)_3_), 3.30 (t, 2H, -NCH_2_CH_2_CH_2_CH_2_Si–), 3.65 (m, 8H, –SCH_2_CH_2_N–).

^19^F-NMR: (CD_3_OD): δ (ppm) −128.3 (s, 2F, CF_3_CF_2_CF_2_CO_2_R), −121.8 (c, 2F, CF_3_CF_2_CF_2_CO_2_R), −82.2 (t, 3F, CF_3_CF_2_CF_2_CO_2_R).

^13^C{^1^H}-NMR: (CD_3_OD): δ (ppm) −4.99 (–Si(CH_3_)), −4.89 (–Si(CH_3_)), 14.5 (–NCH_2_CH_2_CH_2_CH_2_Si–), 15.5 (–SiCH_2_CH_2_S–), 19.3–19.6 (–SiCH_2_CH_2_CH_2_Si–), 22.3 (–NCH_2_CH_2_CH_2_CH_2_Si–), 25.4 (–SiCH_2_CH_2_S-), 28.8 (–SCH_2_CH_2_N–), 33.8 (-NCH_2_CH_2_CH_2_CH_2_Si–), 40.7 (–NCH_2_CH_2_CH_2_CH_2_Si–), 53.6 (–N(CH_3_)_3_), 67.0 (–SCH_2_CH_2_N–).

MS: [M − Cl]^+^ = 1174.4908 Da (calcd. = 1174.4916 Da).

Elemental analysis: C_45_H_98_Cl_4_F_7_N_5_OS_4_Si_3_ (1212.60 g/mol): calcd. = C, 44.57; H, 8.15; Cl, 11.69; F, 10.97; N, 5.78; O, 1.32; S, 10.58; Si, 6.95. Exp. = C, 43.67; H, 8.06; N, 5.45; S, 9.37.

NH_2_G_2_(SC_8_H_4_F_13_)_1_(SO_3_Na)_3_ (**10**)

A solution of PhtG_2_A_4_ (**v**) (0.169 g; 0.292 mmol), 1H, 1H, 2H, 2H-perfluorooctanethiol (0.099 g; 0.263 mmol), DMPA (0.006 g; 0.026 mmol) in THF/MeOH (2:1) was deoxygenated and irradiated at 365 nm for 1h. DMPA (0.026 g; 0.11 mmol) and an aqueous solution of sodium 3-mercapto-1-propanesulfonate (0.187 g; 1.051 mmol) were then added sequentially, with 2 h of irradiation after each addition. After monitoring by ^1^H-NMR, the volatiles were removed under vacuum, and the residue was redissolved in MeOH/H_2_O (6:1) and transfered to an ampoule. Hydrazine hydrochloride (0.145 mL; 4.670 mmol) was added to the solution and heated to 90 °C for 16 h. Finally, the solvents were eliminated and the aqueous solution of the crude product was purified by dialysis (MWCO 100-500 Da). Yield: 82%

^1^H-NMR: (D_2_O): δ (ppm) −0.12 (s, 9H, –Si(CH_3_)), 0.52 (s, 18H, NCH_2_CH_2_CH_2_CH_2_Si–, –SiCH_2_CH_2_CH_2_Si–, –SiCH_2_CH_2_CH_2_S-), 1.28 (m, 6H, –NCH_2_CH_2_CH_2_CH_2_Si–, –SiCH_2_CH_2_CH_2_Si–), 1.48 (m, 6H, –SiCH_2_CH_2_CH_2_S–), 1.59 (m, 2H, –NCH_2_CH_2_CH_2_CH_2_Si–), 1.89 (m, 6H, SCH_2_CH_2_CH_2_SO_3_Na), 2.04 (m, 2H, SCH_2_CH_2_C_6_F_13_), 2.47 (m, 8 H, NCH_2_CH_2_CH_2_CH_2_Si, –SiCH_2_CH_2_CH_2_S–) 2.52 (m, 6H, SCH_2_CH_2_CH_2_SO_3_Na), 2.72 (m, 2H, SCH_2_CH_2_C_6_F_13_), 2.85 (m, 6H, SCH_2_CH_2_CH_2_SO_3_Na).

^19^F-NMR: (CDCl_3_): δ (ppm): −85.8 (m, 3F, CF_3_), −115.5 (m, 2F, –CF_2__ε_), −123.0 (m, 2F, –CF_2__δ_), −124.2 (m, 4F, –CF_2__β_ and –CF_2__γ_), −127.7 (m, 2F, –CF_2__α_).

Elemental analysis: C_42_H_77_F_13_NNa_3_O_9_S_7_Si_3_ (1364.70 g/mol): calcd. = C, 36.97; H, 5.69; N, 1.03; S, 16.44. Exp. = C, 37.09; H, 5.65; N, 1.01; S, 16.47.

NH_2_G_3_(SC_8_H_4_F_13_)_2_(SO_3_Na)_6_ (**11**)

Compound **11** was prepared using the protocol described for **10**, starting from PhtG_3_A_8_ (**vi**) (0.110 g; 0.098 mmol), 1H, 1H, 2H, 2H-perfluorooctanethiol (0.067 g; 0.176 mmol), DMPA (0.005 g; 0.018 mmol), sodium 3-mercapto-1-propanesulfonate (0.125 g; 0.703 mmol), DMPA (0.018 g; 0.070 mmol), hydrazine hydrochloride (0.048 mL; 1.568 mmol). Yield: 87 %

^1^H-NMR: (D_2_O): δ (ppm) −0.12 (s, 21H, –Si(CH_3_)), 0.52 (s, 34H, NCH_2_CH_2_CH_2_CH_2_Si–, –SiCH_2_CH_2_CH_2_Si–, –SiCH_2_CH_2_CH_2_S-), 1.28 (m, 14H, –NCH_2_CH_2_CH_2_CH_2_Si–, –SiCH_2_CH_2_CH_2_Si–), 1.48 (m, 16H, –SiCH_2_CH_2_CH_2_S-), 1.59 (m, 2H, –NCH_2_CH_2_CH_2_CH_2_Si–), 1.89 (m, 14H, SCH_2_CH_2_CH_2_SO_3_Na), 2.04 (m, 2H, SCH_2_CH_2_C_6_F_13_), 2.47 (m, 16 H, NCH_2_CH_2_CH_2_CH_2_Si, –SiCH_2_CH_2_CH_2_S–) 2.52 (m, 14H SCH_2_CH_2_CH_2_SO_3_Na), 2.72 (m, 2H, SCH_2_CH_2_C_6_F_13_), 2.85 (m, 14H, SCH_2_CH_2_CH_2_SO_3_Na).

^19^F-NMR: (CDCl_3_): δ (ppm): −85.8 (m, 3F, CF_3_); −115.4 (m, 2F, –CF_2__ε_), −123.0 (m, 2F, –CF_2__δ_), −124.2 (m, 4F, –CF_2__β_ and –CF_2__γ_), −127.6 (m, 2F, –CF_2__α_)

Elemental analysis: C_87_H_159_F_26_NNa_6_O_18_S_14_Si_7_ (2784.55 g/mol): calcd. = C, 37.53; H, 5.76; N, 0.50; S, 16.12. Exp. = C, 37.61; H, 5.74; N, 0.55; S, 16.09.

## Figures and Tables

**Table 1:** Evaluated reaction conditions in the esterification process.

Base	Equivalents	Temperature	Esterification (%)	Hydrolysis (%)
K_2_CO_3_	1	90	50	50
K_2_CO_3_	1	r.t.	0	0
K_2_CO_3_	0.5	90	65	35
K_2_CO_3_	0.5	r.t.	0	0
NaHCO_3_	1	90	70	30
NaHCO_3_	1	r.t.	0	0
NaHCO_3_	0.5	90	30^1^	25^1^
NaHCO_3_	0.5	r.t.	0	0
NEt_3_	1	90	15	85
NEt_3_	1	r.t.	0	0

^1^ Precursors were also observed.

## Supplementary Materials

The following are available online, Figure S1. ^1^H-NMR spectrum of the incomplete esterification reaction between perfluorhexanoic acid and dendrons with a bromide atom at the focal point. Figure S2. ^19^F-NMR spectrum of the saponification reaction. Figure S3. ^1^H-NMR spectrum of dendron **7**. Figure S4. ^13^C{^1^H}-NMR spectrum of dendron **7**. Figure S5. ^19^F-NMR spectrum of dendron **7**. Figure S6. ^13^C{^19^F}-NMR spectrum of dendron **7**. Figure S7. ESI–TOF of compound **9**.
