# Supplementary material for: New Ionic Carbosilane Dendrons Possessing Fluorinated Tails at Different Locations on the Skeleton
Source: Molecules. 2020 Feb 13;25(4):807. doi: 10.3390/molecules25040807 (PMC7070408; doi:10.3390/molecules25040807)

molecules

SUPPLEMENTARY MATERIALS

Article

New Ionic Carbosilane Dendrons Possessing Fluorinated Tails at Different Locations on the Skeleton

Gabriel Mencia [1,2,3,†], Tania Lozano-Cruz [1,2,3,†], Mercedes Valiente [4], Javier de la Mata [1,2,3,], Jesús Cano [1,3] and Rafael Gómez [1,2,3,*]

[1] Departamento de Química Orgánica y Química Inorgánica, IQAR, Universidad de Alcalá, Campus Universitario, Alcalá de Henares, Madrid 28805, Spain; gabi.men.ber@gmail.com (G.M.); tania.lozano@uah.es (T.L-C.); javier.delamata@uah.es (J.d.l.M.); jesus.cano@uah.es (J.C.)

[2] Networking Research Center on Bioengineering, Biomaterials and Nanomedicine (CIBER-BBN), Madrid 28029 Spain.

[3] Ramón y Cajal Health Research Institute (IRYCIS), IRYCIS, 28034 Spain.

[4] Departamento de Química Analítica, Química Física e Ingeniería Química, IQAR, Universidad de Alcalá Campus Universitario, Alcalá de Henares, Madrid 28805, Spain; mercedes.valiente@uah.es

* Correspondence: rafael.gomez@uah.es; Tel: (+34)-91-885-4685

† These authors contributed equally to this work

Table of content

Figure S1. ^{1}H-NMR spectrum of incomplete esterification reaction between perfluorhexanoic acid and dendrons with a bromide atom at the focal point. ... 2

Figure S2. ^{19}F-NMR spectrum of saponification reaction ... 2

Figure S3. ^{1}H NMR spectrum of dendron **7** ... 3

Figure S4. ^{13}C{^{1}H} NMR spectrum of dendron **7** ... 3

Figure S5. ^{19}F NMR spectrum of dendron **7** ... 4

Figure S6. ^{13}C{^{19}F} NMR spectrum of dendron **7** ... 4

Figure S7. ESI-TOF of compound **9** ... 5

Figure S1. ^{1}H-NMR spectrum of incomplete esterification reaction between perfluorhexanoic acid and dendrons with a bromide atom at the focal point.

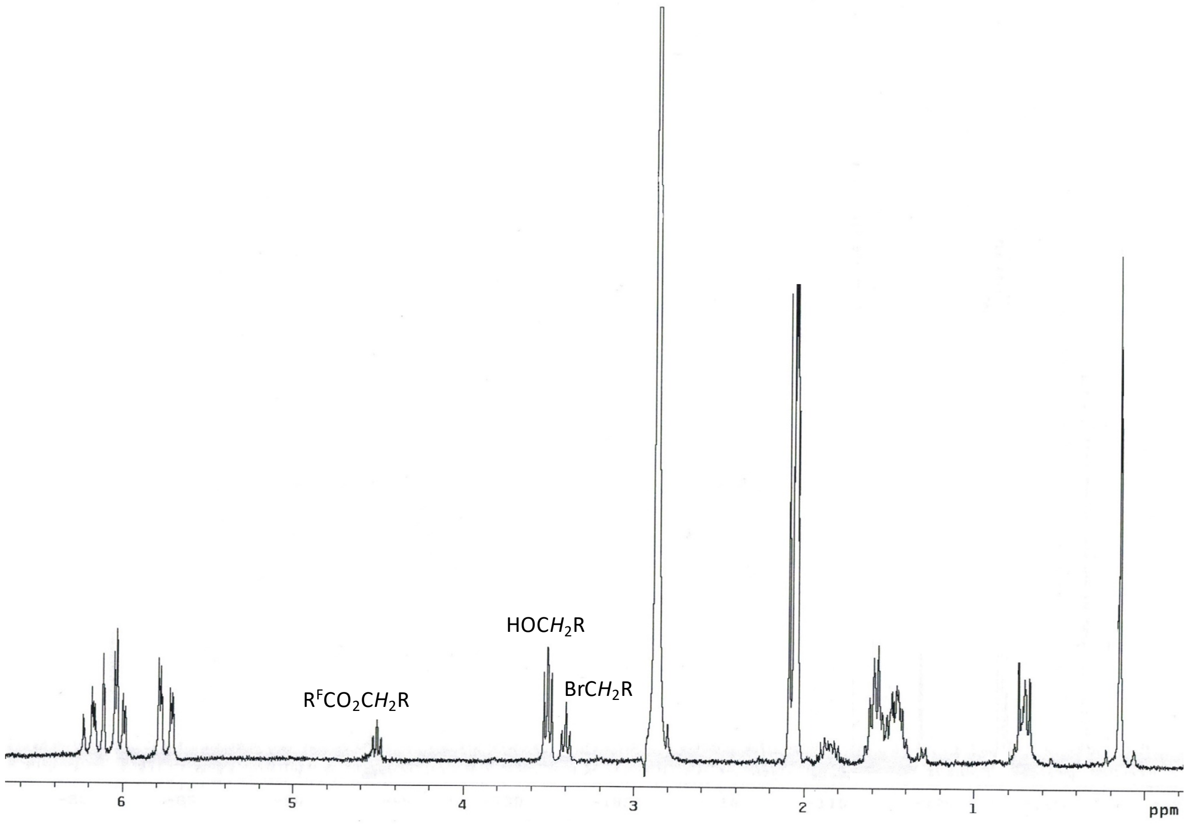

Figure S2. ^{19}F-NMR spectrum of saponification reaction

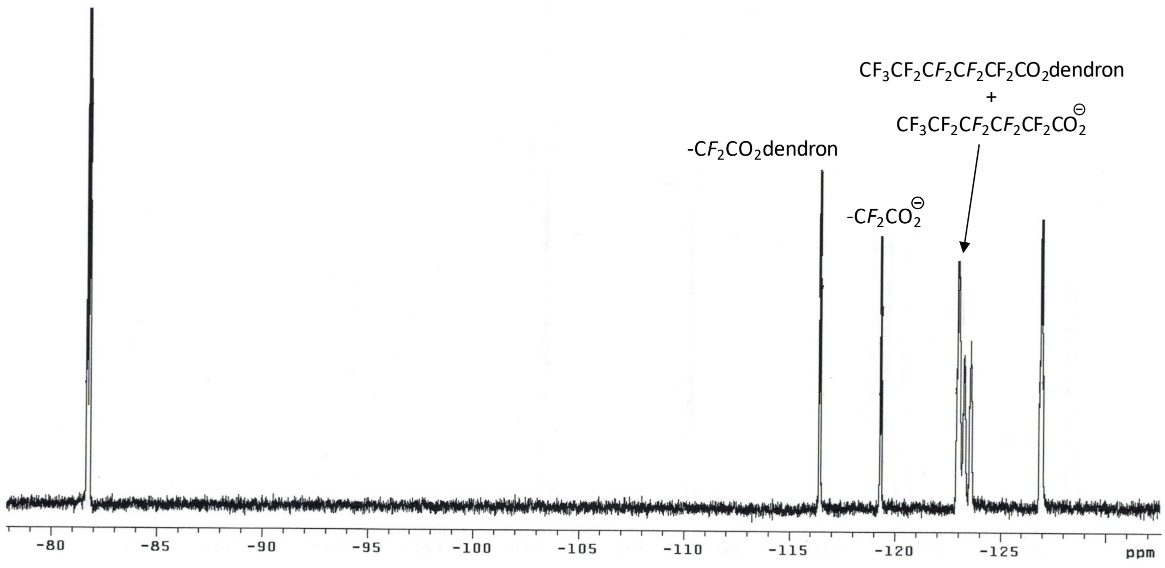

Figure S3. ^{1}H NMR spectrum of dendron **7**

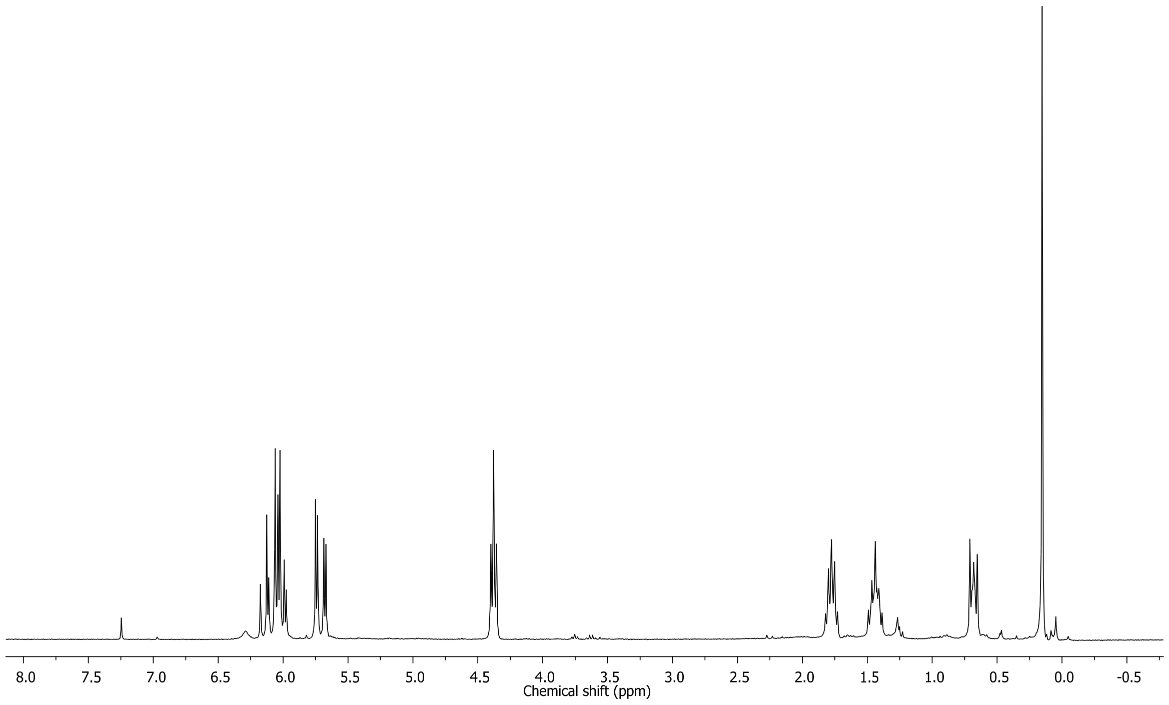

Figure S4. $^{13}C\{^{1}H\}$ NMR spectrum of dendron **7**

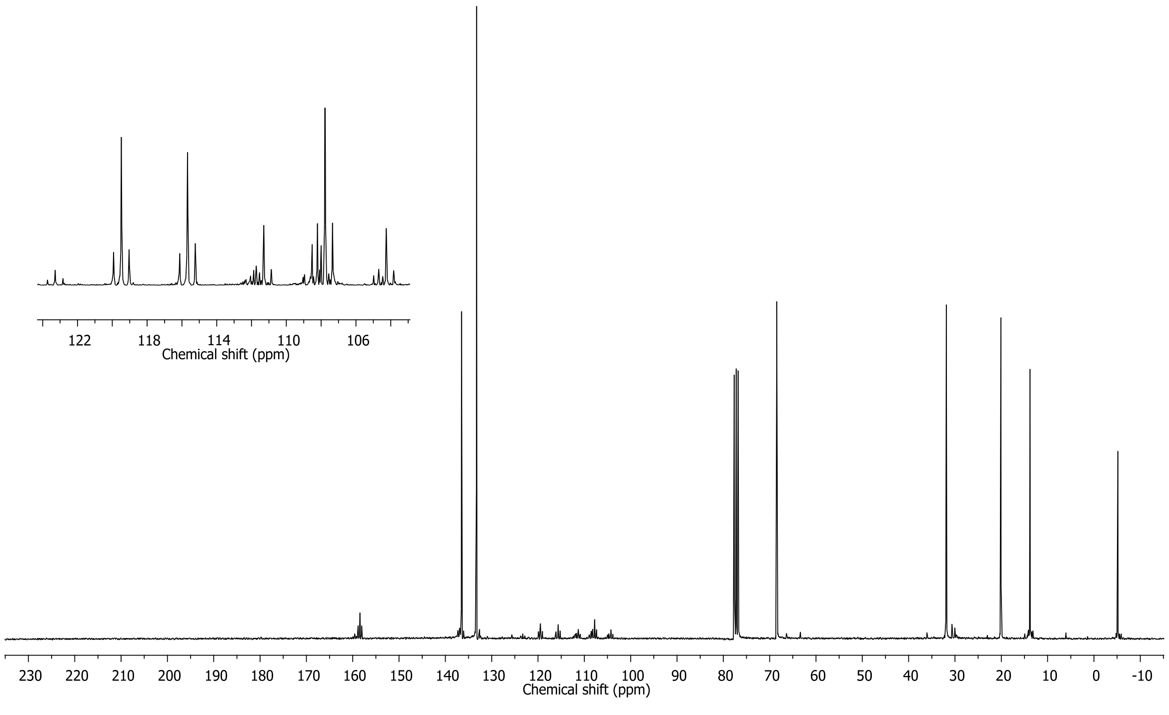

Figure S5. ^{19}F NMR spectrum of dendron **7**

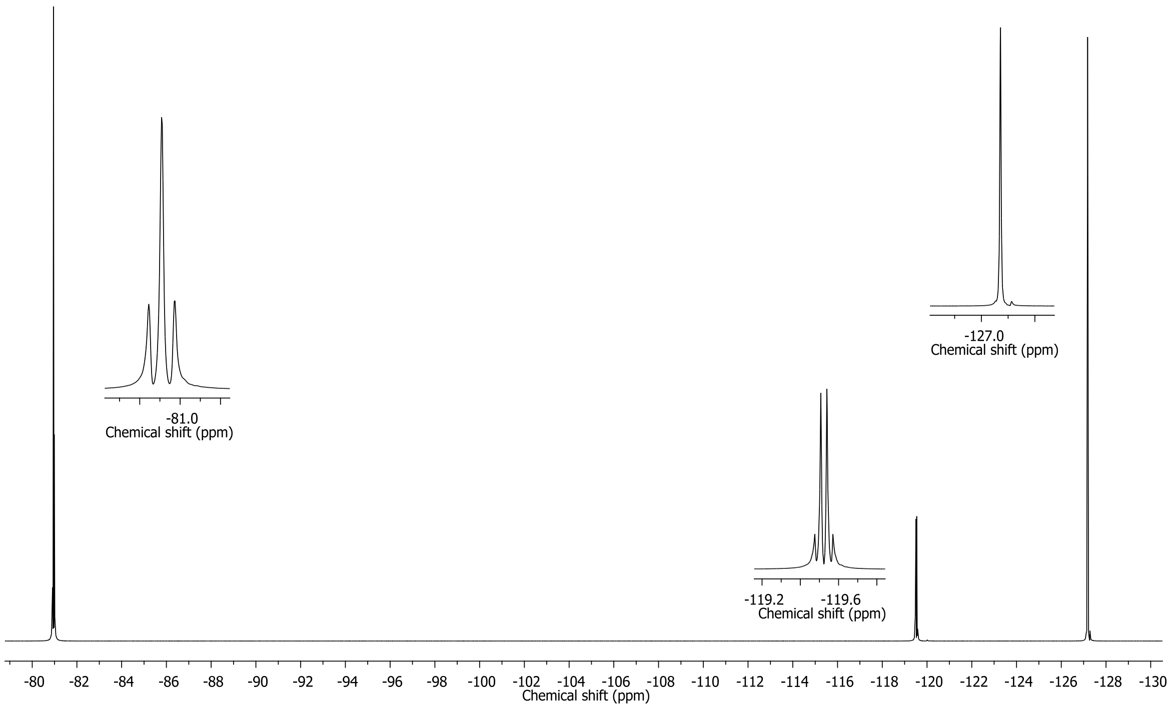

Figure S6. ^{13}C{^{19}F} NMR spectrum of dendron **7**

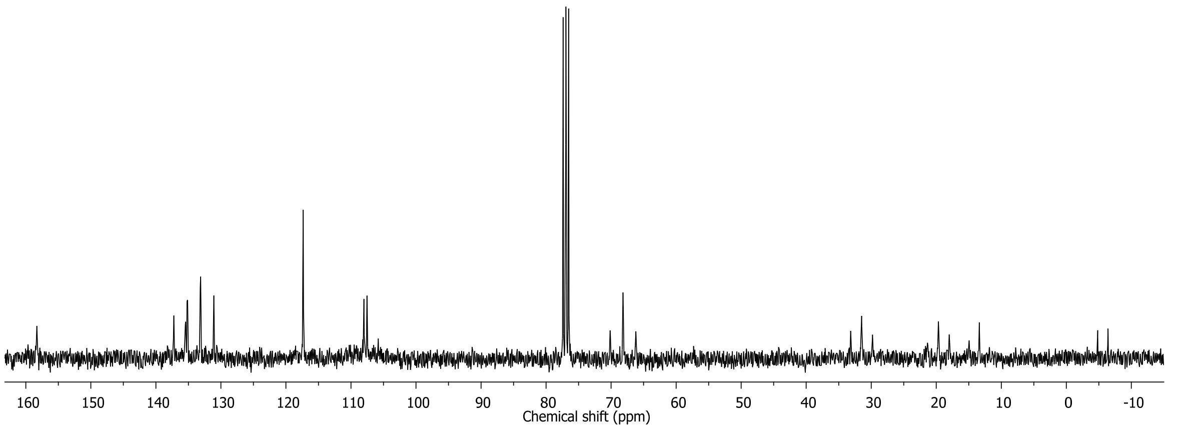

Figure S7. ESI-TOF of compound **9**

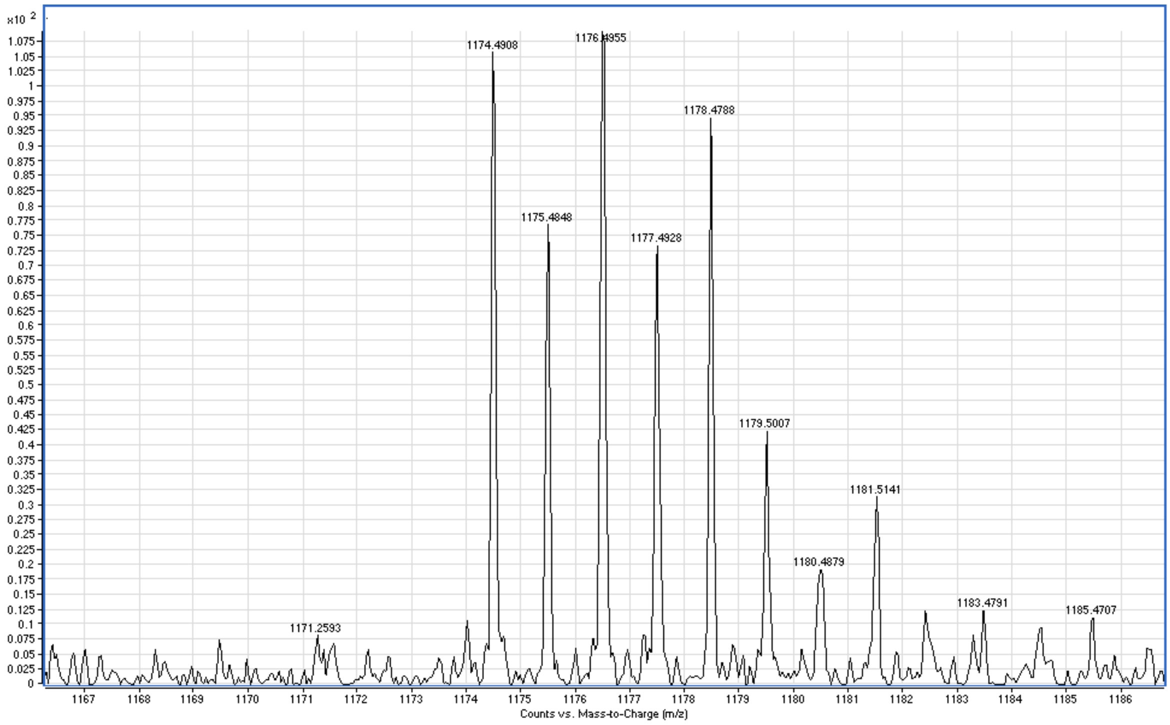

Supplement: Supplementary file 1 [file molecules-25-00807-s001.pdf]
